# How does pea architecture influence light sharing in virtual wheat–pea mixtures? A simulation study based on pea genotypes with contrasting architectures

**DOI:** 10.1093/aobpla/pls038

**Published:** 2012-11-26

**Authors:** Romain Barillot, Didier Combes, Valérie Chevalier, Christian Fournier, Abraham J. Escobar-Gutiérrez

**Affiliations:** 1 LUNAM Université, Groupe Ecole Supérieure d'Agriculture, UPSP Laboratoire d'Ecophysiologie Végétale & Agroécologie, 55 rue Rabelais, BP 30748, F-49007 Angers cedex 01, France; 2 INRA, UR4 P3F, Equipe Ecophysiologie des plantes fourragères, Le Chêne - RD 150, BP 6, F-86600 Lusignan, France; 3 INRA, UMR 759 LEPSE, F-34060 Montpellier, France; 4 SupAgro, UMR 759 LEPSE, F-34060 Montpellier, France

## Abstract

**Background and aims:**

Light interception is a key factor driving the functioning of wheat–pea intercrops. The sharing of light is related to the canopy structure, which results from the architectural parameters of the mixed species. In the present study, we characterized six contrasting pea genotypes and identified architectural parameters whose range of variability leads to various levels of light sharing within virtual wheat–pea mixtures.

**Methodology:**

Virtual plants were derived from magnetic digitizations performed during the growing cycle in a greenhouse experiment. Plant mock-ups were used as inputs of a radiative transfer model in order to estimate light interception in virtual wheat–pea mixtures. The turbid medium approach, extended to well-mixed canopies, was used as a framework for assessing the effects of leaf area index (LAI) and mean leaf inclination on light sharing.

**Principal results:**

Three groups of pea genotypes were distinguished: (i) early and leafy cultivars, (ii) late semi-leafless cultivars and (iii) low-development semi-leafless cultivars. Within open canopies, light sharing was well described by the turbid medium approach and was therefore determined by the architectural parameters that composed LAI and foliage inclination. When canopy closure started, the turbid medium approach was unable to properly infer light partitioning because of the vertical structure of the canopy. This was related to the architectural parameters that determine the height of pea genotypes. Light capture was therefore affected by the development of leaflets, number of branches and phytomers, as well as internode length.

**Conclusions:**

This study provides information on pea architecture and identifies parameters whose variability can be used to drive light sharing within wheat–pea mixtures. These results could be used to build up the architecture of pea ideotypes adapted to multi-specific stands towards light competition.

## Abstract (precis)

Light sharing within virtual wheat-pea mixtures was influenced by the variability of pea’s architectural parameters affecting LAI and height. Light capture was affected by the development of leaflets, number of branches and phytomers and internode length.

## Introduction

Cereal–legume intercropping systems are assumed to provide high and stabilized yields in terms of quantity and quality (Ofori and Stern 1987; Jensen 1996; Corre-Hellou *et al.* 2006), allow lower use of fertilizers and pesticides, and enhance biodiversity conservation (for a review see Malézieux *et al.* 2009). Intercropping benefits result from a trade-off between complementarity (e.g. separate root and canopy areas) and competition processes (for light, water and nutrients) that occur between the component crops. Among the contested resources, solar radiation, and in particular photosynthetically active radiation (PAR), is an important factor driving plant functioning as it provides the energy required for photosynthesis and thus for plant growth. Light capture also drives biological nitrogen fixation by legume species and therefore the autonomy of the intercropping system towards nitrogen resources. Finally, light determines the potential for crop productivity (Loomis and Williams 1963; Goudriaan 1982). Therefore, its sharing in multi-specific stands appeared to be a crucial issue for managing the balance between the component species as well as for determining the final yield of the mixture. It has been proposed that increasing the light interception efficiency (LIE) of intercropping systems could be achieved through: (i) maximizing total light interception by improving the spatial and temporal ground cover and (ii) adequately sharing light between component species by improving their spatial and temporal complementarity (Sinoquet and Caldwell 1995).

Total radiation interception, as well as its sharing among intercropped species, is highly related to the physical structure of the canopy (Ross 1981*b*; Sinoquet and Caldwell 1995), which emerges from the architecture of the individuals growing within the stand (Moulia *et al.* 1998). Individuals' architecture can be described as a collection of subunits called phytomers. After Godin (2000), such a description needs to include (i) an inventory of the plant components (decomposition information), (ii) the topological relationships between these components, and (iii) their geometry, given by the organ shape and spatial position (Godin 2000). Such a multiscale analysis of the canopy structure reveals the importance of plant architectural parameters as underpinning factors determining light interception and sharing within sole and mixed cropped systems.

Contrasting architectural parameters could result from: (i) the genotypic variability of a given species, (ii) the environmental conditions to which plants are exposed during their growth, and (iii) the genotype and environment interactions characterizing the phenotypic plasticity. Architectural plasticity of wheat (*Triticum aestivum* L.) has been described for various cultivars, sowing dates and row orientations, plant densities and nitrogen fertilization regimes (Evers *et al.* 2007; Baccar *et al.* 2011; Dornbusch *et al.* 2011). Plasticity of some architectural components of pea (*Pisum sativum* L.) has also been studied. For instance, Turc and Lecoeur (1997) found that leaf primordium initiation and leaf production of several pea cultivars were coordinated and stable in a large range of environmental conditions. Lateral branching of pea appeared to be dependent not only on environmental conditions such as low temperatures (Jeudy and Munier-Jolain 2005) but also on the genotype and its interactions with plant density (Spies *et al.* 2010). Further, the genetic determination of some architectural parameters of pea is now well documented (for a review see Huyghe 1998). Nevertheless, to our knowledge, the effects of the genetic variability of these architectural parameters on the level of competition for light within cereal–legume intercropping systems have not been reported. Such information would be useful for building up the architecture of pea ideotypes adapted to multi-specific stands.

Recently, virtual plant models have been used to study ideotype architectures in order to optimize light interception and photosynthesis in tomato (Sarlikioti *et al.* 2011) and rice (Zheng *et al.* 2008), and manage light sharing in an integrated legume–weed system (Cici *et al.* 2008). Although virtual plant models are able to take into account the multiscale aspect of plant functioning (stand, plant and organ levels), they have not been used to analyse the relationships between species architecture and light sharing within cereal–legume intercropping systems. The present study therefore focused on wheat–pea mixtures and in particular pea architecture, which has been little described. The aim of the present work was to identify the parameters of pea architecture whose genotypic variability leads to various levels of light sharing within wheat–pea canopies. To this end, we combined the virtual plant approach with an explicit description of the above-ground architecture of six pea genotypes. This allowed us to assess the effects of contrasting pea architectures on light sharing within mixtures.

### Theory of light sharing in well-mixed canopies

In monocrop stands, light capture is well described by applying the Beer–Lambert law (Monsi and Saeki 1953) on homogeneous canopies. This approach is based on the assumption that the canopy can be described as a turbid medium where leaf area index (LAI) and mean leaf inclination are the main information needed. In our study, the turbid medium approach was used as a framework for assessing the effect of LAI and mean leaf inclination on light sharing within a range of intercrop architectures. Indeed, some authors extended this approach to the case of well-mixed intercropping systems (Rimmington 1985; Sinoquet and Bonhomme 1991). Under such conditions, LIE of a species *i* in a mixture of *N* components is given by (1)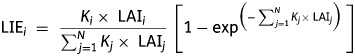 where *K_i_* and LAI*_i_* are the extinction coefficient and leaf area index of species *i*. The extinction coefficients are derived from the mean leaf inclination of species (Sinoquet *et al.* 2000). Light sharing in the mixture of two species is therefore given by the ratio of the LIE of one species over the total light interception of the canopy. The contribution of species 1 to light capture of a bi-specific mixture is thus estimated by (2)
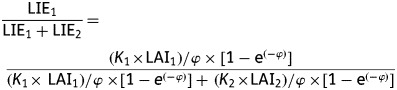
 with 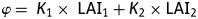.

**Fig. 1:**
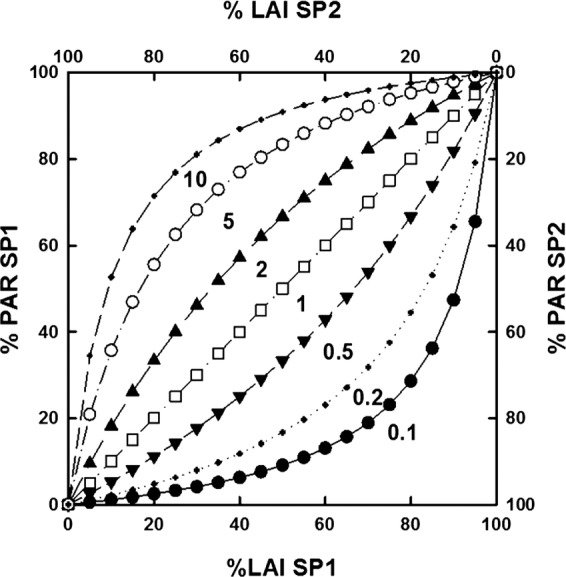
**Light sharing in a well-mixed mixture of two species (SP1 and SP2) as a function of their contribution to the total LAI.** Theoretical isolines are shown for different ratios of the species extinction coefficients (*K*). *K*_SP1_/*K*_SP2_ ranged from 0.1 to 10.

Simplifying, (3)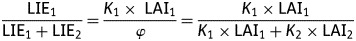 Considering 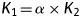, we obtain (4)
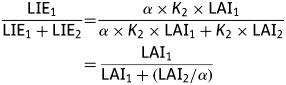
 Figure 1 shows the relative light interception by species 1 and 2 as a function of their contribution to the LAI of the mixture. For well-mixed canopies, theoretical isolines of light interception have been derived from equation (4) for different values of *α*, i.e. ratios of *K*_1_/*K*_2_. On the other hand, heterogeneous canopies would not follow these theoretical isolines, meaning that light sharing is not mainly determined by the species LAI and leaf inclination. Indeed, a component species can exhibit different height, leading to a vertical arrangement of the canopy. In this case, information on the vertical structure of the canopy is needed for estimating light sharing between component species (Sinoquet 1993; Barillot *et al.* 2011).

## Methods

### Growing conditions and plant material

Six winter pea cultivars (*P. sativum*) were grown in 2 L individual plastic pots, spaced 10 cm apart, in a greenhouse from 24 March 2011 to 7 June 2011 in Angers, France (47°27′N, 00°32′W). Air temperature in the greenhouse ranged from 15 to 28 °C, water supply was adapted to maintain soil moisture content around 15 % (w/w). Individual pots were filled with soil containing 47 mg of NO_3_^−^ kg^−1^. To ensure pea nodulation, each plant was inoculated with a solution of *Rhizobium leguminosarum* P221 at 2.2 × 10^9^ bacteria L^−1^. Pea genotypes included four semi-leafless cultivars: Lucy, James, AOPH10 and 886/01 (HR type), and two leafy cultivars: China and US13 (Table 1). Eight plants of each pea cultivar were arranged according to a monofactorial experimental design in two randomized complete blocks, which were surrounded by non-measured plants in order to avoid border effects.

**Table 1:** **Description of pea cultivars.** Contribution of stipules, leaflets and stems to the maximum green area is also indicated for each pea cultivar.

Pea cultivar	Leaf type	hr/HR^a^	Last digitizing date (DD)	Organ contribution to the green area (%)		
				Stipules	Leaflets	Stems
China	Leafy	hr	1310	47 ± 2.8	45 ± 2.3	8 ± 0.9
US13	Leafy	hr	1445	46 ± 0.7	49 ± 0.9	5 ± 0.2
Lucy	Semi-leafless	hr	1565	91 ± 0.6	–	9 ± 0.6
James	Semi-leafless	hr	1445	89 ± 0.8	–	11 ± 0.8
AOPH10	Semi-leafless	hr	1630	91 ± 1.0	–	9 ± 1.0
886/01	Semi-leafless	HR	1890	91 ± 0.8	–	9 ± 0.8

^a^Pea cultivars of hr type are not sensitive to photoperiod, whereas cultivar 886/01 shows high response to photoperiod (HR type).

### Three-dimensional digitizing of pea plants and virtual reconstructions

**Fig. 2:**
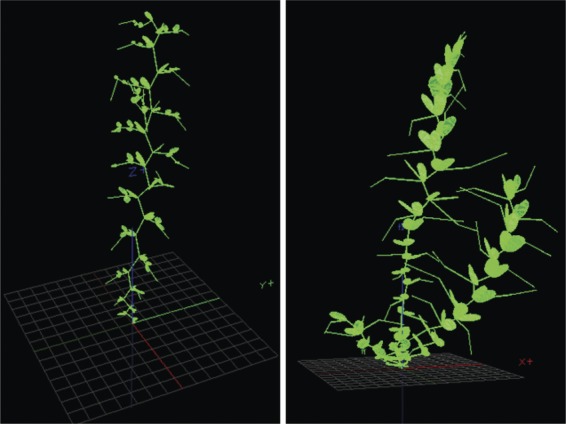
**Horizontal views of some reconstructed pea cultivars.** Left: the semi-leafless cultivar 886/01; right: the leafy cultivar US13, both at 1000 DD.

Pea cultivars were digitized twice a week during their growing cycle from 200 growing degree-days (DD) after emergence (base temperature 0 °C) until physiological maturity. Physiological maturity of pea ranged from 1310 to 1890 DD (Table 1), which represented 21 dates of measurement for the latest cultivars. Details on the digitizing procedure and virtual plant reconstruction are given in Barillot *et al.* (2011). Briefly, magnetic digitizing was carried out by using a three-dimensional (3D) digitizer (3Space Fastrak, Polhemus Inc., Colchester, VT, USA). Spatial coordinates as well as each phytoelement orientation (apart from tendrils which were not digitized) were collected and encoded as Multiscale Tree Graphs. Data from digitizing were then imported into the Openalea platform (Pradal *et al.* 2008) where plant mock-ups were digitally reconstructed for each digitized plant (Fig. 2). Digitizing from 800 DD was done on phytomers located above the half-height of stems (for main stems and branches), assuming that by this time lower phytomers had completed their growth (Turc and Lecoeur 1997). Non-measured phytomers were reconstructed from their previous digitizing, thus reducing the duration of digitizing. Computations of LAI, height and architectural parameters of the pea genotypes were thus performed on the virtual mock-ups.

### Calculation of light interception

#### Radiative model

Light interception estimates are based on a nested radiosity model (Chelle and Andrieu 1998). Computations were performed considering only diffuse radiations according to the Uniform Overcast Sky radiation distribution (Moon and Spencer 1942). Diffuse radiations were approximated by a set of light sources on a sky vault discretized in 20 solid angle sectors using the spherical coordinates, including 5 zenith angles (18°, 36°, 54°, 72°, 90°) and 4 azimuths. Light interception was computed for each direction and then integrated over the sky vault by summing up the 20 directional values.

#### Estimation of light sharing within virtual wheat–pea mixtures

Light sharing was computed for virtual wheat–pea mixtures at four digitization dates: 300, 600, 1240 and 1560 DD. Wheat (*T. aestivum*) mock-ups were obtained from a dynamic but non-plastic 3D architectural model of wheat development (Fournier *et al.* 2003). The data set was derived from an experiment of Bertheloot *et al.* (2009), where wheat plants (cultivar Caphorn) were grown in field conditions under low-nitrogen fertilization at a density of 250 plants m^−2^. In the present study, wheat mock-ups were generated at the same stages of development as pea and then merged with pea reconstructions in the Openalea platform (Pradal *et al.* 2008). Inter-row spacing of virtual mixtures was 17 cm with a final density of 125 plants m^−2^ for wheat and 45 plants m^−2^ for pea, i.e. 50 % of each crop optimal density used in local agricultural practices (Corre-Hellou *et al.* 2006). Species were mixed within each row.

### Statistical analysis

Data analyses were performed by using exploratory data analysis and variance analysis. Pea cultivars were compared by using the Tukey's honestly significant difference (HSD) test. The significance threshold was fixed at the 0.05 probability level for all statistical tests. Statistical analyses were performed with SAS 9.2 (SAS Institute, Cary, NC, USA) and R (R Development Core Team 2011).

The variable phytomer appearance (defined by the number of phytomers visible, i.e. emergence of the stipules from the apex) was fitted with Schnute's non-linear model (Schnute 1981) using the least-square method. The model is written as (5)
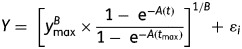
 where *Y* is the phytomer appearance variable; parameters *A* and *B* implicitly define the shape of the curve; *t*_max_ is the last value of the time (*t*) domain for which the model is fitted; and parameter *y*_max_ is the value of *Y* at *t*_max_. Parameters were optimized using the Levenberg–Marquardt iterative method with automatic computation of the analytical partial derivatives.

The first derivatives of Schnute adjustments were used in order to estimate the rates of phytomer appearance of the pea cultivars.

## Results and discussion

### Light sharing within wheat–pea mixtures

To dynamically analyse the effects of the architectural parameters of pea on light sharing in a mixture, we first assessed, for each contrasting genotype, the relationships between light interception and LAI, foliage inclination and plant height. These basic parameters contribute to plant architecture and thus to canopy structure (Sinoquet and Caldwell 1995). Details on wheat and pea mock-ups used for each simulation time are summarized in Table 2. Leaf area index of pea plants ranged from 0.06 m^2^ m^−2^ for cultivar Lucy at 300 DD to 3.44 m^2^ m^−2^ for 886/01 at 1560 DD. Foliage inclination of pea cultivars, ranging from 20.8° to 55°, was more planophile than that of wheat (64.5° on average throughout the growing cycle). At the first stage of development (300 DD), the height of wheat and pea was similar (11.3 cm for wheat and 13.2–17.5 cm for pea genotypes). In contrast, pea cultivars largely overtopped wheat plants from 600 DD. This was particularly the case for the cultivar China, which reached 127 cm at 1240 DD.

**Table 2:** LAI, foliage inclination and plant height of wheat and pea mock-ups used to simulate light sharing in virtual mixtures.

Species	Genotype	Growing degree-day (°C day)			
		300	600	1240	1560
Leaf area index (m^2^ m^−2^)					
Pea	China	0.11	0.81	1.81	
Pea	US13	0.13	0.79	2.42	
Pea	Lucy	0.06	0.29	1.29	
Pea	James	0.08	0.34	1.12	
Pea	AOPH10	0.07	0.37	1.96	2.03
Pea	886/01	0.08	0.39	2.48	3.44
Wheat	Caphorn	0.13	0.83	1.00	0.46
Foliage inclination (°)					
Pea	China	20.8	30.2	40.9	
Pea	US13	32.3	32.9	46.6	
Pea	Lucy	44.5	43.3	48.7	
Pea	James	38.0	39.3	45.2	
Pea	AOPH10	37.8	38.7	49.7	49.8
Pea	886/01	41.7	38.6	51.1	55.0
Wheat	Caphorn	65.5	67.0	64.3	61.0
Plant height (cm)					
Pea	China	17.5	70.7	127.1	
Pea	US13	14.4	45.5	94.7	
Pea	Lucy	13.2	32.7	83.6	
Pea	James	13.8	36.1	81.3	
Pea	AOPH10	13.9	32.0	84.2	87.1
Pea	886/01	14.6	31.3	70.4	86.3
Wheat	Caphorn	11.3	16.0	65.2	69.1

#### Effects of LAI and foliage inclination on light sharing

**Fig. 3:**
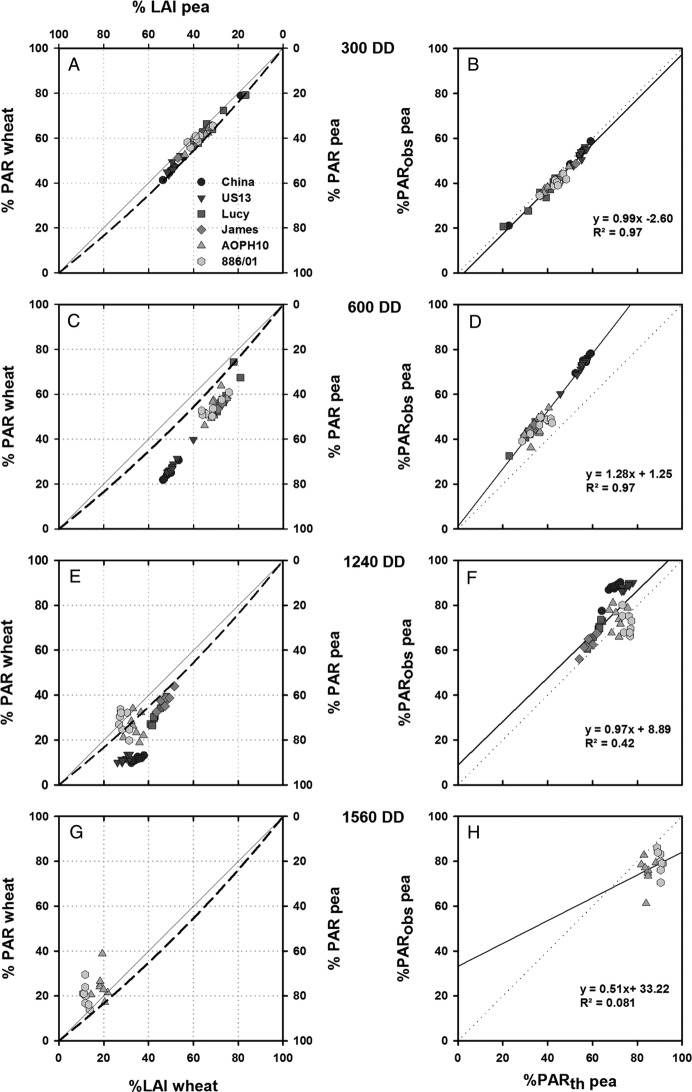
**Light sharing within virtual wheat–pea mixtures at 300, 600, 1240 and 1560 DD.** Relative contributions of the species to the LAI of the mixture and to PAR interception are shown in (A), (C), (E) and (G). Theoretical isolines, based on the ratio of extinction coefficients of the species, are represented by dotted lines. Deviations between the estimated values of light interception of pea and the theoretical isoline are given in (B), (D), (F) and (H) where the equation and coefficient of determination *R*^2^ of linear regressions are also shown. For each simulation date (300, 600, 1240 and 1887 DD), computations were performed on eight plants of each pea cultivar.

The relationships between species' relative contribution to light interception and LAI of virtual intercrops at four stages of development are shown in Fig. 3 (left column). Based on the observed foliage inclination, the ratio of the species extinction coefficient was computed in order to perform a theoretical estimation of light sharing for each simulation (dotted lines). These theoretical values of light sharing, based on the turbid medium approach, were thus estimated assuming that canopies were well-mixed. Contrasting deviations were observed between computed and theoretical values of the species' relative contribution to light interception within the virtual mixtures (Fig. 3, right column). In the early stages of development (Fig. 3A and C), a wide range of relative LAI emerged from the variability in plant architecture between the studied genotypes. This variability in relative LAI resulted in an equivalent variability in LIE by each of the two species. At 300 DD (Fig. 3A), computed values of light sharing were close to the theoretical isoline estimated for well-mixed canopies (Fig. 3B, slope of the linear regression = 0.99, *R*^2^ = 0.97). At this early stage of development (species LAI ranging from 0.06 to 0.13 m^2^ m^−2^, Table 2), light sharing therefore appeared to be strongly dependent on the architectural parameters that contributed to the LAI and foliage inclination of each species. At 600 DD (Fig. 3C), pea genotypes appeared more efficient for light interception compared with the previous stage of development. This was particularly the case for the leafy cultivars China and US13, which exhibited similar amounts of LAI to wheat (0.81, 0.79 and 0.83 m^2^ m^−2^, respectively, Table 2) and which captured 70–75 % of light intercepted by the mixture. Nevertheless, computations of light sharing strongly deviated from the theoretical isoline (Fig. 3D, slope of the linear regression = 1.28, *R*^2^ = 0.97), meaning that light sharing was not only determined by the architectural parameters that contribute to the species LAI and foliage orientation.

After 1200 DD (Fig. 3E and F), LAI of the pea genotypes (1.12–3.44 m^2^ m^−2^, Table 2) was higher than that of wheat (1.00–0.46 m^2^ m^−2^, Table 2). Pea therefore captured >50 % of the radiation intercepted by the mixture. At 1240 DD (Fig. 3E), three groups of pea genotypes are distinguishable: (i) the cultivars Lucy and James, which were close to the theoretical isoline, (ii) ‘AOPHA10’ and ‘886/01’ cultivars, vertically distributed around the isoline and (iii) the cultivars China and US13, exhibiting a strong efficiency for light capture. The two last groups, for which the contribution to total LAI exceeded 60 %, showed divergences between computed values of light sharing and the theoretical approach that assumes well-mixed canopies (Fig. 3F, slope of the linear regression = 0.97, *R*^2^ = 0.42). Thus, at this late stage of development, the architectural parameters that determine the LAI and the foliage inclination of these cultivars do not only determine light sharing between the component species. Similar conclusions could be drawn for the simulations made at 1560 DD (Fig. 3G and H). Indeed, the two late semi-leafless cultivars, AOPH10 and 886/01, constituted 81 and 88 % of the whole LAI and intercepted 80 and 75 % of incident light, respectively. These two cultivars were less efficient at capturing light in regard to their relative contribution to the LAI of the mixture. This could be related to a combination of architectural parameters that lead to intra-specific foliage clumping (Nilson 1971) due to high values of LAI. Indeed at 1560 DD, cultivars AOPH10 and 886/01 exhibited an LAI of 2.03 and 3.44 m^2^ m^−2^, respectively, whereas wheat LAI was 0.46 m^2^ m^−2^.

The above results show that when canopy closure and species interactions begin, the level of light sharing was not only determined by the species LAI and foliage inclination as can be estimated for well-mixed canopies. Light sharing within such mixtures is therefore also related to architectural parameters that lead to heterogeneous canopies such as contrasted vertical profiles of leaf area or height between component species (Sinoquet and Caldwell 1995).

#### Effects of pea genotype height on light sharing

**Fig. 4:**
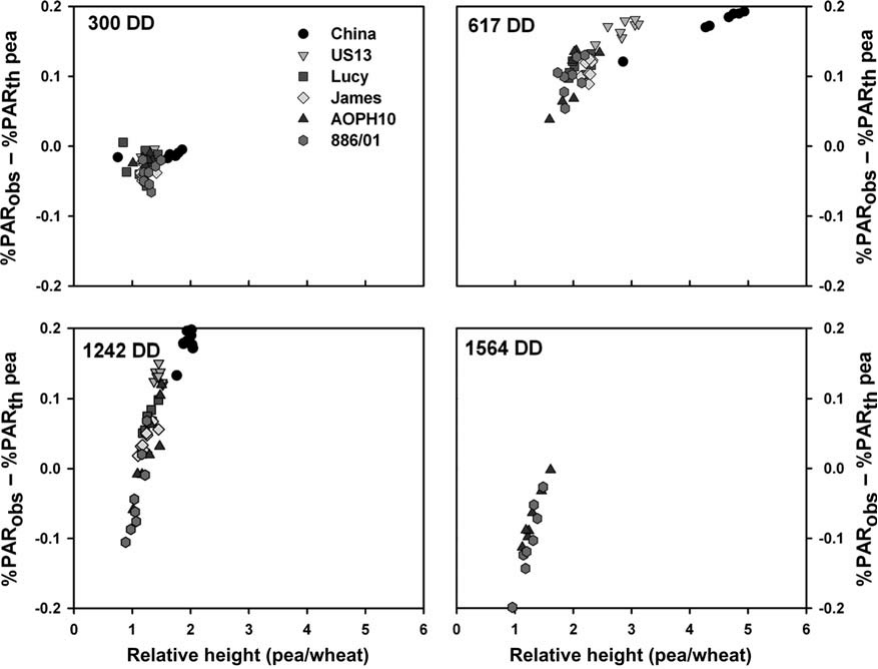
Relationship between the species height ratio and the deviations between computed and theoretical (based on the ratio of extinction coefficients of the species) values of light interception.

Deviations between computed values of light sharing and the theoretical approach, which assumes well-mixed canopies, were then studied in regard to the relative height of the component species (wheat/pea height, Fig. 4). As expected from the previous analysis, no clear relationship could be established between the species height ratio and the deviations of estimated values of light interception at 300 DD. On the other hand, these deviations appeared to be exponentially related to the species height for the simulations made at 600, 1240 and 1560 DD. Indeed, deviations between computed values of light interception and the theoretical approach were strongly related to the species relative height for wheat/pea ratios ranging from 1 to 2, whereas, for higher values, the effect of pea height on light sharing remained constant. These results are consistent with the study of Sinoquet and Caldwell (1995), who showed that the difference in light interception between two species in a mixture increased when the relative height deviates from the 1:1 line. The present results also demonstrate that this relationship is preserved for concomitant variations of plant LAI and height.

### Characterization of the architectural parameters of pea genotypes

In the first step of this study, we assessed the impact of the pea genotypes on light sharing within virtual mixtures through basic descriptors, i.e. LAI, foliage inclination and plant height. The second step of this work then consisted in a characterization of the above-ground architecture of the pea genotypes in order to analyse the contribution of the architectural parameters to light interception.

#### Branching

The number of branches and the number of phytomers per branch produced by the pea cultivars are given in Table 3. In a few cases, cultivars China, US13 and Lucy developed one branch with 2.8–8.0 phytomers at their maximal development. Most of the plants belonging to cultivars ‘James’ and ‘AOPH10’ produced one branch early in their growing cycle (between 300 and 600 DD). However, branches developed by cultivar James did not develop beyond 6.0 (±2.16) phytomers, contrasting with cultivar AOPH10 (25.7 ± 1.5). Cultivar 886/01 was the most branching cultivar with one to three branches developed since 600 DD and composed of 12.4–23.5 phytomers. Branches were initiated from the basal part of the main stem (i.e. on the primordia of vestigial leaves) except for ‘China’ whose branches were mainly initiated between the 12th and 14th phytomer. Our results show that the pea genotypes exhibited different branching capacities, although lateral branches were less developed compared with field-grown spring (Jeudy and Munier-Jolain 2005; Spies *et al.* 2010) and winter cultivars (unpublished personal results). It has been well documented that branching is strongly determined by the environment. Winter cultivars generally produce a large number of lateral branches due to frost damage to the apex of the main stem that occurs during the growing cycle (Jeudy and Munier-Jolain 2005). In our study, the greenhouse growing conditions prevented frost damage to the apex so that its dominance on axillary buds was maintained (Cline 1991). Spies *et al.* (2010) also showed that basal branching in spring pea cultivars was dependent on the sowing density. Indeed, independently of the trophic aspects of light competition, plant density also modifies the quality of light within the canopy (Varlet-Grancher *et al.* 1993; Escobar-Gutiérrez *et al.* 2009) and in particular the red/far red ratio, which has been reported to control branching (Ballaré and Casal 2000). The branching ability of pea cultivars is therefore a plastic architectural parameter that strongly impacts light interception as the development of a new vegetative axis modifies the amount of foliar area as well as its spatial distribution.

**Table 3:** **Number of branches and number of phytomers per branch of pea cultivars at each simulation date.** B1, B2 and B3 point out the chronological emission of branches. Mean values are given ± SD (*n* = 8 for each cultivar).

Growing degree-day	Pea cultivar	Range of the number of branches	Mean number of phytomers per branch		
			B1	B2	B3
300	China	0			
	US13	0			
	Lucy	0			
	James	[0–1]	3.0 ± 0 (*n* = 5)		
	AOPH10	[0–1]	3.0 ± 0 (*n* = 4)		
	886/01	0			
600	China	[0–1]	2.5 ± 0.5 (*n* = 2)		
	US13	0			
	Lucy	0			
	James	[0–1]	5.7 ± 2.0 (*n* = 6)		
	AOPH10	[0–2]	9.5 ± 0.5 (*n* = 6)	5.0 ± 0 (*n* = 1)	
	886/01	[0–2]	7.4 ± 1.9 (*n* = 5)	3.7 ± 0.7 (*n* = 5)	
1240	China	[0–1]	2.8 ± 0.4 (*n* = 4)		
	US13	[0–1]	8.0 ± 0 (*n* = 1)		
	Lucy	[0–1]	3.0 ± 0 (*n* = 1)		
	James	[0–1]	6.0 ± 2.16 (*n* = 3)		
	AOPH10	[0–2]	22.8 ± 1.3 (*n* = 6)	6.0 ± 0 (*n* = 1)	
	886/01	[1–3]	18.6 ± 4.1 (*n* = 8)	14.2 ± 2.0 (*n* = 6)	10.0 ± 2.3 (*n* = 4)
1560	AOPH10	[0–1]	25.7 ± 1.5 (*n* = 6)		
	886/01	[1–3]	23.5 ± 3.8 (*n* = 8)	16.9 ± 6.4 (*n* = 7)	12.4 ± 5.1 (*n* = 5)

#### Phytomer appearance

**Fig. 5:**
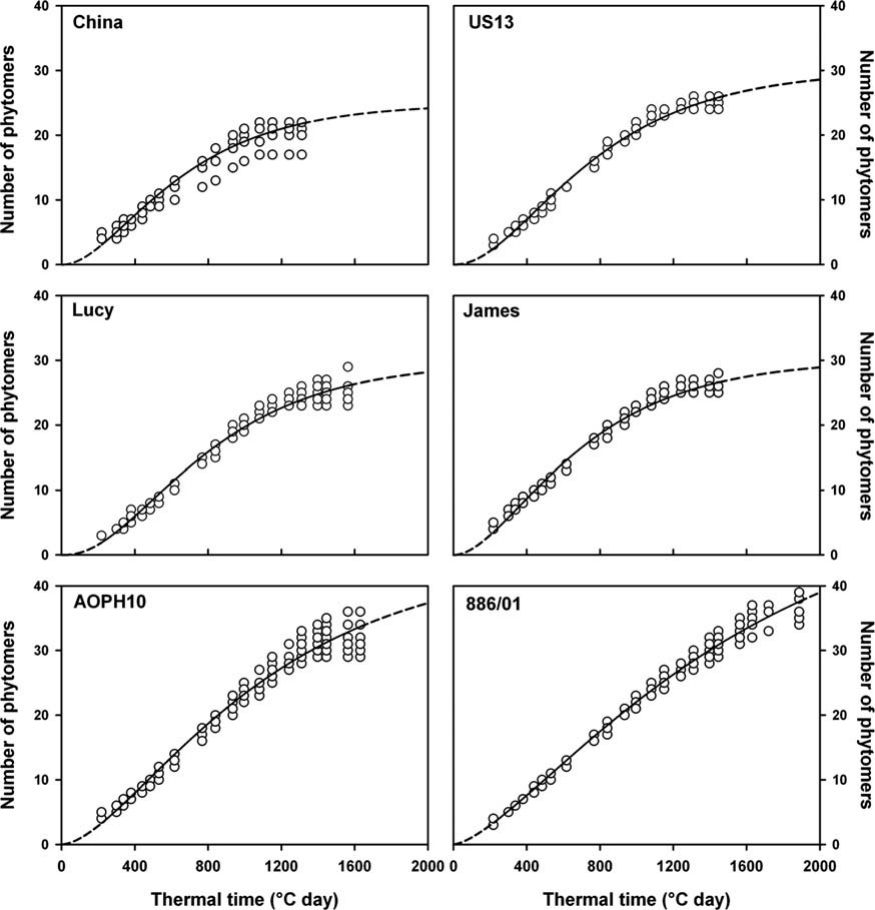
**Phytomer appearance for the pea cultivars during the growing cycle.** Values are indicated for main stems only. The fitting of Schnute's functions is shown by a solid line or by a dotted line when extrapolated (*n* = 8 for each cultivar).

Dynamics of phytomer appearance were measured on main stems, assuming that phyllochrons of branches were similar (Jeudy and Munier-Jolain 2005). Phytomer appearance on the main stems followed a contrasting range of sigmoid-type dynamics (Fig. 5) among the evaluated cultivars. These dynamics were fitted with the Schnute function (Table 4). The cultivar China reached its maximum number of phytomers (21, see Table 4) at 1080 DD, while cultivars ‘US13’, ‘Lucy’ and ‘James’ reached 25 phytomers at 1240 DD. Owing to a long period of phytomer production, cultivars ‘AOPH10’ and ‘886/01’ reached 32 and 37 phytomers at 1445 and 1890 DD, respectively. Maximum rates of phytomer appearance were reached in the earlier stages of development (between 350 and 530 DD) and ranged from 0.025 to 0.029 phytomer DD^−1^ (Table 4). ‘China’ and ‘James’ were the earliest cultivars and reached their maximum rate of phytomer appearance at 350 and 360 DD, respectively. On the other hand, ‘886/01’ was the latest cultivar and exhibited its highest rate of phytomer production at 530 DD. The kinetics of phytomer appearance are usually estimated by a mean value over the growing cycle (e.g. Turc and Lecoeur 1997; Bourion *et al.* 2002). However, our results show that pea phytomers are not produced at a constant rate. In this case, Schnute's non-linear regressions appeared to be well suited to estimate the rates of phytomer appearance. Rates of phytomer initiation (plastochron) and appearance (phyllochron) (Lyndon 1998) characterize the development of the vegetative axes (main stems and branches). Phytomer appearance is therefore a key parameter of plant architecture that determines (i) the dynamics of foliar development and (ii) the height reached by the vegetative axis (stack of phytomers), thus affecting the ability of a plant to capture light.

**Table 4:** Parameters (*A*×10^−3^, *B* and *y*_max_) of Schnute adjustments made on cultivars' kinetics of phytomer emission. Indicated values are the mean ± SD of eight individual adjustments made for each cultivar. Goodness of fit is also given by RMSE values. Cultivars with the same letter are not significantly different (Tukey's HSD test, *α* = 0.05). First derivative of the adjustments provided the maximum rate of phytomer emission.

Parameter	Cultivar					
	China	US13	Lucy	James	AOPH10	886/01
Kinetics of phytomer emission (Schnute adjustments)						
*A* (×10^−3^)	2.14 ± 0.34^a^	1.89 ± 0.28^a^	1.89 ± 0.34^a^	1.94 ± 0.26^a^	1.18 ± 0.22^b^	0.80 ± 0.22^b^
*B*	0.47 ± 0.08^dc^	0.44 ± 0.05^dc^	0.39 ± 0.05^d^	0.49 ± 0.05^bc^	0.57 ± 0.05^ab^	0.65 ± 0.08^a^
*y*_max_	20.6 ± 1.6^d^	24.6 ± 0.5^c^	24.8 ± 1.1^c^	25.6 ± 0.7^c^	32.9 ± 2.1^b^	37.6 ± 1.6^a^
RMSE	0.69	0.72	0.75	0.70	0.81	0.47
Maximal rate of phytomer emission						
Time (DD)	350	440	490	360	480	530
*V*_max_ (phytomer °C day^−1^)	0.026	0.027	0.026	0.029	0.028	0.025

#### Organ final length and vertical distribution

The contribution of stipules, leaflets and stems to the maximum green area reached by plants was also estimated for each cultivar (Table 1). Leaves (stipules and leaflets) accounted for >90 % of the green area whatever the cultivar. For leafy cultivars, this area was similarly composed of stipules and leaflets. The contribution of stems to the green area appeared to be constant whatever the genotype (8–11 %), except for the leafy cultivar US13 whose stems constitute 5 % of the cultivar green area.

**Fig. 6:**
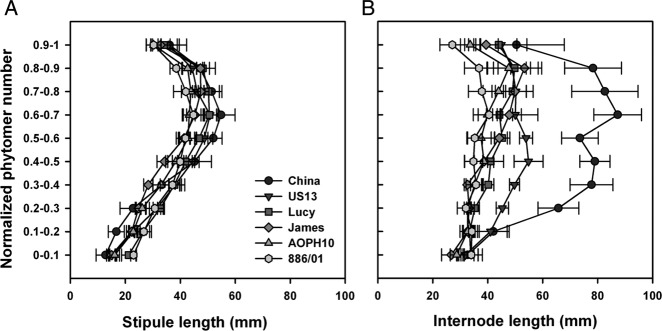
**Vertical distribution of stipule (A) and internode (B) length.** Indicated values are the final lengths reached by organs as a function of the normalized phytomer number of the main stem (*n* = 8 for each pea cultivar).

The vertical distribution of the main vegetative organs is shown in Fig. 6. The vertical distribution of stipule final length did not appear to be dependent on the cultivar (Fig. 6A) but was reported to be rather sensitive to environmental factors such as water stress deficits (Lecoeur *et al.* 1995). The final length reached by stipules appeared to be dependent on their position along the stem as the distribution of stipule length followed triangular profiles as described by Lecoeur (2005). The longest stipules (50 mm) were located between the normalized phytomer numbers 0.6 and 0.8. For leafy cultivars China and US13, the vertical distribution of leaflet length was also dependent on their position on the stem (data not shown). Maximum values of leaflet length (45 mm) were found at the middle of the main stem. The spatial distribution and dimensions of the vegetative organs significantly affect the efficiency of light interception (Niinemets *et al.* 2004*a*, *b*; Pearcy *et al.* 2005). For pea plants, the size and localization of stipules and leaflets would indeed determine the amount of foliar area exposed to solar radiation.

Among the tested cultivars, the vertical distribution of internode length was nearly uniform (Fig. 6B), although maximum values tend to be reached on the upper part of the stem (between normalized phytomer numbers 0.5 and 0.8). Within this interval, the leafy cultivars displayed longer internodes than semi-leafless cultivars; this was particularly the case for ‘China’ whose internodes exceeded 75 mm in length. The cultivars AOPH10 and 886/01 developed the shortest internodes on average (37.0 and 35.0 mm, respectively). ‘Lucy’ and ‘James’ were intermediate cultivars with mean internode length reaching 41.5 and 40.0 mm, respectively. Internode lengths and their spatial distribution are also key parameters of pea architecture determining plant height and leaf area density, and consequently the ability to capture light.

#### Stipule and leaflet inclination

**Fig. 7:**
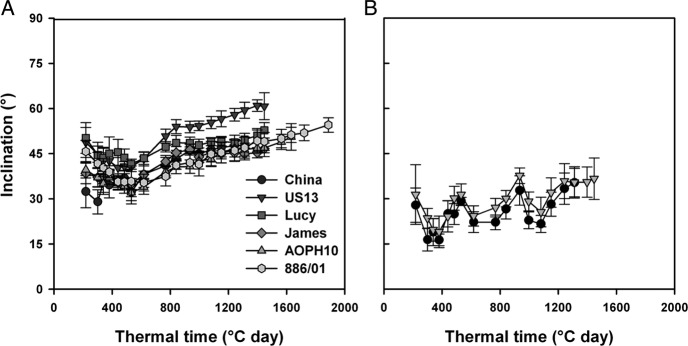
**Dynamics of stipule (A) and leaflet (B) inclination during the growing cycle.** Values are indicated for main stems only (*n* = 8 for each pea cultivar).

Inclination of stipules located on main stems ranged from 30 to 60° among cultivars but was not constant over time as all cultivars showed a slight increase in stipule inclination from 600 DD (Fig. 7A). At the end of the growing cycle, the inclination of stipules ranged from 45° to 55°, except for ‘US13’ which showed more erect stipules reaching 60°. Leaflets of the two leafy cultivars China and US13 were more planophile than stipules as their inclination ranged from 16° to 37° (Fig. 7B). These results on foliage inclination were consistent with previous studies conducted on legume species (Ross 1981*a*; Barillot *et al*. 2011). As mentioned before, leaf inclination has been described as an architectural parameter that contributes to the efficiency of plants in intercepting light (Sinoquet and Andrieu 1993).

### Relationships between pea architecture and light sharing

Our results allowed us to distinguish three groups of development among the tested cultivars. The first group is composed of the leafy cultivars (China and US13) which exhibited a rapid vertical and leaf area growth. The second group is constituted by the late cultivars (AOPH10 and 886/10, late hr and HR-type, respectively) which also developed a large leaf area. The third group is composed of cultivars with low developmental rates, ‘Lucy’ and ‘James’, which displayed the smallest leaf area and height.

These contrasting pea architectures were then virtually mixed with wheat mock-ups obtained from data where wheat was grown under low nitrogen supply (Bertheloot *et al.* 2009). The level of available nitrogen is obviously a major factor impacting the species growth. However, increasing the nitrogen availability in a cereal–legume mixture enhances the dominance of the cereal species over the legume (e.g. Corre-Hellou *et al.* 2006; Naudin *et al.* 2010). Cereal–legume mixtures are therefore usually grown under low-nitrogen conditions that are close to the data from which the mock-ups were derived. Within open canopies, our results showed that light sharing was mainly related to the architectural parameters that composed the LAI and leaf inclination. Thus, the earliness and the development of leaflets exhibited by the leafy cultivars as well as a large number of phytomers and branches (late semi-leafless cultivars) led to strong competitive abilities for light capture. When canopy closure and competition between component species started, light sharing within the virtual mixtures also appeared to be strongly dependent on the species height ratio. The vertical dominance of pea was due to (i) the rapid development of long internodes for leafy cultivars and (ii) the stack of numerous phytomers on stems for late semi-leafless cultivars. In addition, the dominance of pea genotypes was reinforced by the planophile property of their leaves compared with wheat. However, as very few differences were observed between leaf inclinations of cultivars, this architectural parameter does not appear to be a major factor explaining the contrasting levels of light sharing observed in the different wheat–pea mixtures.

## Conclusions and forward look

In the present study, the virtual plant approach was used as a means to provide a suitable framework for the assessment of the effects of the architectural parameters of pea on light sharing within wheat–pea mixture. Nevertheless, the present approach did not account for plant plasticity, i.e. above and below-ground feedbacks between the mixed species were not taken into account. Moreover, wheat and pea mock-ups were derived from data obtained in mono-specific conditions and pea plants were staked, although this can be representative of the field conditions as wheat plants generally support pea stems. However, these mock-ups were not aimed to be representative of the development of species grown in a field mixture. These virtual plant models were intended to generate a large range of physical structures reflecting contrasting canopies which can be found in multi-specific stands. Therefore, pea plants that exhibited contrasting architectures (stemming from the different cultivars and stages of development) were virtually mixed with a given wheat architecture in order to compare their efficiency for light interception in relation to their architectural features. The present study highlights levers of pea architecture that are determinant parameters driving light sharing. These parameters were mainly involved in the composition of the species LAI and height. Competitive pea ideotypes for light capture therefore exhibited (i) a large foliar area, through the presence of leaflets and/or the development of a larger number of branches and phytomers, and/or (ii) a vertical dominance due to the stack of numerous phytomers and the development of long internodes. To drive the interspecific competition, the developmental dynamics of these architectural parameters must also be taken into account in regard to the other components of the mixture. In addition, we identified contrasting architectural parameters among the tested genotypes, associated to variability in their earliness/lateness that would, in field conditions, lead to different levels of competition for light in the mixture. Further studies should be conducted on supplementary genotypes and growing conditions in order to reveal additional categories of architectural development and assess their effects on light sharing. At the present state-of-the-art, it is still complex to assess the optimal level of light sharing within intercropping systems. However, the present results could help to identify/design suitable cultivars/ideotypes for intercropping systems towards light sharing, in particular because of the genetic progress which has already identified several genes driving pea architecture.

## Sources of funding

This research is supported by ‘La Région Pays de la Loire’, France through a Ph.D. fellowship to R.B. The research of D.C. and A.E.-G. is partially funded by ‘La Région Poitou-Charentes’, France.

## Contributions by the authors

All the authors contributed to a similar extent overall.

## Conflicts of interest statement

None declared.
